# A De Novo Splicing Mutation of *SRP72* in Bone Marrow Failure Syndrome Type 1: Case Report and Review of the Literature

**DOI:** 10.1002/mgg3.70168

**Published:** 2025-12-31

**Authors:** Wang Xiangwen, Zhang Duo, Hao Wenjing, Hou Hui

**Affiliations:** ^1^ Department of Pediatric Xiaogan First People's Hospital Xiaogan China; ^2^ Baotou Medical College Inner Mongolia University of Science and Technology Baotou China; ^3^ Department of Science and Education Chifeng Hospital Chifeng China; ^4^ Department of Pediatric Hematology Inner Mongolia Autonomous Region People's Hospital Hohhot China

**Keywords:** bone marrow failure syndrome type 1, case report, minigene, *SRP72*

## Abstract

**Background:**

*SRP72*‐associated hereditary bone marrow failure syndrome type 1 (BMFS1) has recently been described and only six families have been reported so far. BMFS1 is an autosomal dominant condition characterized by early‐onset aplastic anemia or pancytopenia in some patients and adult‐onset myelodysplasia in others. This paper presents the clinical and genetic characteristics of a rare case of hereditary bone marrow failure syndrome 1 (BMFS1) and explores its pathogenesis.

**Methods:**

Blood samples and clinical data were collected from the proband and his biological parents. Next‐generation sequencing (NGS) was employed to sequence the genes associated with the proband, and the identified variants were subsequently confirmed via Sanger sequencing. Additionally, minigene splicing assays were conducted to assess the functional alterations of *SRP72*.

**Results:**

A new splicing variant, c.1502+1G>A, was identified in the *SRP72* gene through gene sequencing, and this finding was confirmed by Sanger sequencing. Neither parent carried this mutation. Minigene splicing assays revealed an insertion of two bases (AG) at the mRNA level (r.1503‐2_1503‐1insAG), potentially resulting in a premature stop codon (p.Leu502ValfsTer14). According to ACMG guidelines, the variant is classified as “Likely pathogenic”. The c.1502+1G>A mutation in *SRP72* is implicated as the potential cause of BMFS1 in this child.

**Conclusions:**

Our study identified a novel classical splicing mutation, marking the first report of BMFS1 in China. This case broadens the spectrum of pathogenic variants associated with the *SRP72* gene and expands our understanding of its phenotypic manifestations. It also serves as a typical example for early diagnosis and appropriate treatment of BMFS1.

## Graphical abstract

This study reports a rare case of bone marrow failure syndrome type 1 (BMFS1) caused by a novel de novo splicing mutation (c.1502+1G>A) in the *SRP72* gene. The 6‐year‐old patient presented with aplastic anemia and pancytopenia. Genetic analysis identified the mutation, which was absent in both parents, confirming its de novo origin. Mini‐gene assays demonstrated that the mutation leads to abnormal mRNA splicing, resulting in a frameshift mutation and premature stop codon (p.Leu502ValfsTer14). This is the first reported case of BMFS1 in China, expanding the mutation spectrum of *SRP72* and highlighting the importance of genetic testing in diagnosing BMFS1. The findings provide new insights into the molecular mechanisms of BMFS1 and underscore the role of *SRP72* in bone marrow failure.

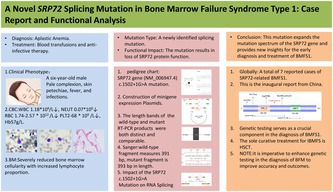

**Abbreviations**
AA: aplastic anemia
BMFS1: bone marrow failure syndrome type 1
HSCT: hematopoietic stem cell transplantation
NGS: next generation sequencing
SRP: signal recognition particle

## 1 Introduction

Inherited bone marrow failure syndromes (BMFS) comprise a heterogeneous group of life‐threatening diseases, including Fanconi anemia (FA), dyskeratosis congenita (DC), DiamondBlackfan anemia (DBA), and Shwachman‐Diamond syndrome (SDS) (Dokal et al. 2022; Kim et al. 2022). Their hallmark features include single‐lineage cytopenia or pancytopenia, congenital malformations, bone marrow hematopoietic failure, and increased susceptibility to tumors (Bluteau et al. 2018; Li and Bledsoe 2023). BMFS1 (OMIM: #614675) is a rare form of BMFS caused by mutations in the *SRP72* gene (OMIM: #602122) (Kirwan et al. 2012), often necessitating hematopoietic stem cell transplantation (HSCT) therapy.

The *SRP72* gene encodes the 72‐kD subunit of the signal recognition particle (SRP), a ribonucleoprotein with dual functions (Kellogg et al. 2022; Iakhiaeva et al. 2005). SRP mediates the targeting of proteins, including both membrane and secretory proteins, to the translocon at the surface of the endoplasmic reticulum and protects their mRNA from degradation (Nagai et al. 2003; Faoro and Ataide 2021). SRP is composed of six different protein subunits (SRP9, SRP14, SRP19, SRP54, SRP68, and *SRP72*) and a long single noncoding RNA (7SL RNA). It is divided into two domains: the S‐domain and the Alu‐domain (Becker et al. 2017). The S‐domain, which recognizes the signal sequence of the ribosome nascent chain and binds to the signal recognition particle receptor (SR), comprises the central region of 7S RNA, along with the peptides SRP19, SRP54, and a heterodimer of SRP68/*SRP72*. The Alu‐domain, responsible for elongation retardation, consists of a heterodimer of SRP9/SRP14 and the 5′ and 3′ terminal regions of 7S RNA (Faoro and Ataide 2021; Becker et al. 2017). If SRP is abnormal, it may lead to targeted protein degradation or mislocalization, which can eventually result in disease. Indeed, SRP dysfunction has been reported to be associated with many diseases, such as severe congenital neutropenia type 8, autosomal dominant (OMIM: #618752), related to *SRP54* (OMIM: #604857) (Carapito et al. 2017; Fan et al. 2023); severe congenital neutropenia type 10, autosomal recessive (OMIM: #620534), related to *SRP68* (OMIM: #604858) (Saettini et al. 2020; Schmaltz‐Panneau et al. 2021), as well as idiopathic inflammatory myopathy, infections, and cancer (Kellogg et al. 2022).

*SRP72*, the largest α‐solenoidal protein, forms an unusually stable heterodimer with SRP78 and is an essential functional component of the SRP (Becker et al. 2017). Mutations in the *SRP72* gene are causative for familial aplastic anemia and increase the risk of acute myeloid leukemia (Kirwan et al. 2012).

BMFS1 is an autosomal dominant inherited disease characterized by bone marrow failure, manifesting as aplastic anemia and/or myelodysplasia, and associated with hearing and ear abnormalities (such as deafness and labyrinthitis). BMFS1 is very rare, with only six families reported as of March 2024 (Bluteau et al. 2018; Kirwan et al. 2012; Similuk et al. 2022). However, due to insufficient knowledge and the nonspecific clinical phenotype of reported cases, the estimated number of misdiagnosed or undiagnosed patients is difficult to precisely determine.

The phenotype, prognosis, and molecular spectrum of causative *SRP72* variants are insufficiently understood, leading to difficulties in patient management. To broaden the molecular and clinical understanding of BMFS1, we report the phenotype of a 6‐year‐old boy with a novel splicing pathogenic variant in the *SRP72* gene, identified using a panel sequencing associated with bone marrow failure.

## 2 Materials and Methods

### 2.1 Ethical Compliance

This study was conducted with the approval of the Ethics Committee of the Inner Mongolia Autonomous Region People's Hospital, China. Informed consent forms were signed by study participants before blood collection. This study was conducted under the guidelines of the Declaration of Helsinki.

### 2.2 Next‐Generation Sequencing and Sanger Sequencing

Panel sequencing (associated with bone marrow failure) was conducted by MyGenostics Inc., Beijing, China. The tested gene list is shown in Table 1. Informed consent for genetic analysis was obtained from the proband's parents. Peripheral blood samples from the proband and his parents were collected, and DNA was extracted using the QIAamp DNA Mini Kit (Qiagen, Shanghai, China) according to the manufacturer's instructions. Genomic DNA was fragmented to approximately 200–300 bp by enzyme digestion (MyGenostics Inc., Beijing, China). Standard libraries were prepared using the DNA Sample Prep Reagent Set, and the target sequencing library was captured using the H044p1 capture kit (MyGenostics Inc., Beijing, China). The enriched libraries were sequenced on the DNBSEQ‐T7 sequencer for paired‐end reading of 150 bp. After sequencing, low‐quality reads (< 80 bp) were filtered using Cutadapt software (http://code.google.com/p/cutadapt/). The clean reads were then mapped to the UCSC hg19 human reference genome, and variants of SNP and InDel were detected using the Sentieon software (https://www.sentieon.com/) parameter driver. The called variants were annotated using multiple databases, including 1000 Genomes, ESP6500, GnomAD, EXAC, and HGMD, and predicted by tools such as SIFT, PolyPhen‐2, MutationTaster, REVEL, and SpliceAI, using ANNOVAR software (http://annovar.openbioinformatics.org/en/latest/) (Wang et al. 2010). The pathogenicity of the variants was assessed according to the American College of Medical Genetics and Genomics (ACMG) guidelines (Richards et al. 2015).

**TABLE 1:** The tested gene list.

*ABCG5*	*CSF3R*	*FLT3*	*LSG1*	*PRKCD*	*RPL31*	*RPS27A*	*STIM1*
*ABCG8*	*CTC1*	*FMC1‐LUC7L2*	*LUC7L2*	*PRPF40B*	*RPL32*	*RPS27L*	*STN1*
*ABL1*	*CTCF*	*FOXP3*	*MAD2L2*	*PRPF8*	*RPL34*	*RPS28*	*STON1*
*ABL2*	*CTLA4*	*FYB1*	*MAP2K1*	*PSMB6*	*RPL35*	*RPS29*	*STON1‐GTF2A1L*
*ACD*	*CTNNA1*	*G6PC3*	*MASTL*	*PTEN*	*RPL35A*	*RPS3*	*SUZ12*
*ACP5*	*CUX1*	*GALE*	*MBD4*	*PTPN11*	*RPL36*	*RPS3A*	*TBPL1*
*ACSL6*	*CXCR4*	*GAR1*	*MDM4*	*PTPRJ*	*RPL36A*	*RPS4X*	*TERC*
*ACTN1*	*CYCS*	*GATA1*	*MECOM*	*RAD21*	*RPL36A‐HNRNPH2*	*RPS4Y1*	*TERF1*
*ADA*	*DAXX*	*GATA2*	*MECOM‐AS1*	*RAD51*	*RPL36AL*	*RPS4Y2*	*TERT*
*ADA2*	*DCLRE1B*	*GATA3*	*MLH1*	*RAD51C*	*RPL37*	*RPS5*	*TET2*
*ADAMTS13*	*DDX41*	*GFI1*	*MPIG6B*	*RAG1*	*RPL37A*	*RPS6*	*THPO*
*ALAS2*	*DIAPH1*	*GFI1B*	*MPL*	*RAG2*	*RPL38*	*RPS6KA1*	*TINF2*
*ANKRD26*	*DKC1*	*GGCX*	*MRE11*	*RASGRP1*	*RPL39*	*RPS6KA2*	*TLR8*
*ARHGEF1*	*DNAJC21*	*GNAS*	*MSH2*	*RB1*	*RPL39L*	*RPS6KA3*	*TNFSF13B*
*ARID2*	*DNMT3A*	*GNB1*	*MSH6*	*RBBP6*	*RPL3L*	*RPS6KA4*	*TP53*
*ASXL1*	*DRG1*	*GNE*	*MYB*	*RBM8A*	*RPL4*	*RPS6KA5*	*TPM4*
*ATG2B*	*EED*	*GP1BA*	*MYBL2*	*RFWD3*	*RPL41*	*RPS6KA6*	*TPP1*
*ATM*	*EFL1*	*GP1BB*	*MYC*	*RFX5*	*RPL5*	*RPS6KB1*	*TRPM7*
*ATP5IF1*	*EIF6*	*GP9*	*MYH9*	*RFXANK*	*RPL6*	*RPS6KB2*	*TSR2*
*ATRX*	*ELANE*	*GRK3*	*MYSM1*	*RFXAP*	*RPL7*	*RPS7*	*TTC7A*
*BCL2L1*	*ELAVL1*	*GSKIP*	*NAF1*	*RIT1*	*RPL7A*	*RPS8*	*TUBB1*
*BCOR*	*EP300*	*GTPBP4*	*NBEAL2*	*RPL10*	*RPL8*	*RPS9*	*U2AF1*
*BCORL1*	*EPCAM*	*HAX1*	*NBN*	*RPL10A*	*RPL9*	*RPSA*	*U2AF2*
*BLM*	*EPO*	*HJV*	*NCOR2*	*RPL11*	*RPLP0*	*RSL24D1*	*UBA52*
*BOD1L1*	*ERCC4*	*HOXA11*	*NF1*	*RPL12*	*RPLP1*	*RTEL1*	*UBE2T*
*BRAF*	*ERCC6L2*	*IDH1*	*NHP2*	*RPL13*	*RPLP2*	*RUNX1*	*UHRF1*
*BRCA1*	*ESCO2*	*IDH2*	*NIPBL*	*RPL13A*	*RPS10*	*SAMD9*	*UMODL1*
*BRCA2*	*ETV6*	*IFNG*	*NLRP1*	*RPL14*	*RPS11*	*SAMD9L*	*USB1*
*BRCC3*	*EZH2*	*IKZF1*	*NMD3*	*RPL15*	*RPS12*	*SBDS*	*USP1*
*BRINP3*	*FAAP100*	*IL2RA*	*NOP10*	*RPL17*	*RPS13*	*SETBP1*	*VPS13B*
*BRIP1*	*FAAP20*	*IL2RB*	*NOTCH1*	*RPL17‐C18orf32*	*RPS14*	*SF1*	*VPS45*
*CALR*	*FAAP24*	*IL7R*	*NPM1*	*RPL18*	*RPS15*	*SF3A1*	*VWF*
*CASP10*	*FAN1*	*IRF1*	*NRAS*	*RPL18A*	*RPS15A*	*SF3B1*	*WAS*
*CBL*	*FANCA*	*ITGA2*	*ORAI1*	*RPL19*	*RPS16*	*SH2B3*	*WDR48*
*CBLB*	*FANCB*	*ITGA2B*	*PALB2*	*RPL21*	*RPS17*	*SLC34A1*	*WRAP53*
*CBLIF*	*FANCC*	*ITGB3*	*PARN*	*RPL22*	*RPS18*	*SLFN14*	*WRN*
*CD3G*	*FANCD2*	*ITPA*	*PAX5*	*RPL23*	*RPS19*	*SLX4*	*WT1*
*CDC42*	*FANCE*	*JAGN1*	*PCNT*	*RPL23A*	*RPS19BP1*	*SMC1A*	*XRCC2*
*CDKN2A*	*FANCF*	*JAK2*	*PDGFA*	*RPL24*	*RPS2*	*SMC3*	*ZAP70*
*CEBPA*	*FANCG*	*JAK3*	*PDGFB*	*RPL26*	*RPS20*	*SPRED1*	*ZCCHC8*
*CENPS*	*FANCI*	*JARID2*	*PDS5B*	*RPL27*	*RPS21*	*SRC*	*ZFPM1*
*CENPS‐COTR*	*FANCL*	*KDM6A*	*PHF6*	*RPL27A*	*RPS23*	*SRP54*	*ZRSR2*
*CENPX*	*FANCM*	*KIT*	*PMS2*	*RPL28*	*RPS24*	*SRP72*	*ZSWIM4*
*CHEK2*	*FAS*	*KMT2A*	*PNP*	*RPL29*	*RPS25*	*SRSF2*	*STAT3*
*CIITA*	*FASLG*	*KMT2C*	*POT1*	*RPL3*	*RPS26*	*STAG2*	*PRKACG*
*CREBBP*	*FBXW7*	*KRAS*	*PRF1*	*RPL30*	*RPS27*	*STAT1*	*FLI1*

The potential pathogenic variants identified in the proband were validated by Sanger sequencing using a forward primer F1 (5′‐AAGCTCTGAAACGTGGAGTTG‐3′) and a reverse primer R1 (5′‐CCTAAGTTGGATCTATCGCCG‐3′) designed with Primer Premier v5.0 software. The PCR products were further purified and sequenced using an Applied Biosystems ABI3730XL genetic analyzer (Applied Biosystems, Foster City, CA, USA).

### 2.3 Minigene Splicing Assay

To verify the splicing effects of c.1502+1G>A in the *SRP72* (NM_006947.4) gene, a minigene splicing assay was performed in vitro. The minigene region, including exon 14 to exon 16, was amplified from the gDNA of the wild type using a forward primer (5′‐AAGCTTGGTACCGAGCTCGGATCCCCAAAATCTCCTGCTCATTTGTCCTTGA‐3′) and a reverse primer (5′‐TTAAACGGGCCCTCTAGACTCGAGCCTTGTTCCTTTGGTTGACTATCTCCAG‐3′). The amplified products were cloned into the pMini‐CopGFP vector to construct the wild‐type plasmid. The mutant‐type plasmid was created using the mutagenesis primers *SRP72*‐MT‐F (5′‐CAGAGAAAGCCAAAGCaTATCCTTTTGATTGTTATTCCTTACAGC‐3′) and *SRP72*‐MT‐R (5′‐TGCTTTGGCTTTCTCTGGATCTACAAGTGAGT‐3′). The recombinant plasmids of both the wild‐type and mutant‐type were respectively transfected into HEK293T cells using Lipofectamine 3000 (Invitrogen) following the manufacturer's instructions.

Total RNA was extracted from cells cultured for 48 h using TRIzol reagent (Invitrogen, USA). RT‐PCR was conducted using the primer pair MiniRT‐F (5′‐GGCTAACTAGAGAACCCACTGCTTA‐3′) and MiniRT‐R (5′‐CCTTGTTCCTTTGGTTGACTATCTC‐3′). The size of the amplified PCR fragments was analyzed by 1% agarose gel electrophoresis, and the predicted transcripts were sequenced by Sanger sequencing.

## 3 Results

### 3.1 Clinical Presentation

The proband, a 6‐year‐old boy, was admitted to the Department of Pediatric Hematology at Inner Mongolia Autonomous Region People's Hospital due to a “pale face for over two weeks and the presence of petechiae and ecchymosis on the skin for six days.” His birth history was unremarkable, being delivered via Caesarean section at full term. His nonconsanguineous parents are healthy, with no family history of hereditary disease. He was born without any congenital anomalies and has no significant medical history.

At 6 years old, he was diagnosed with aplastic anemia. He was noted to have pancytopenia with leukopenia(1.18–2.39*10^9^/L↓), neutropenia(0.07–0.63*10^9^↓), reduced red cell count (1.74–2.57 * 10^12^ /L↓), thrombocytopenia (2–68 * 10^9^ /L↓), and decreased hemoglobin levels (57–81 g/L).

**FIGURE 1:**
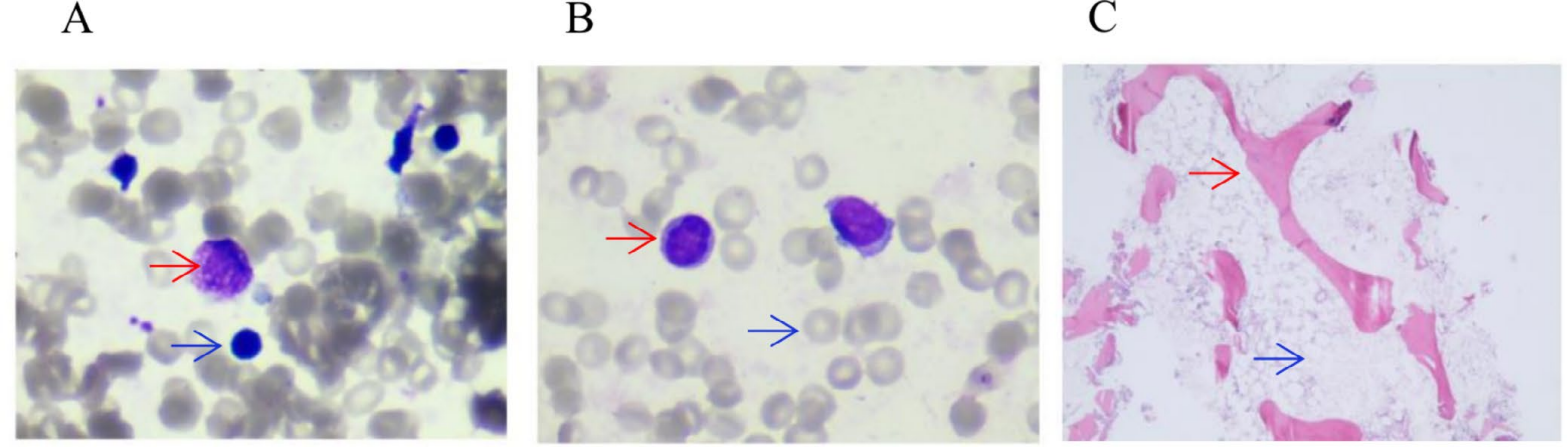
Myelopoietic cells were significantly reduced, lymphocyte and plasma cells were increased, megakaryocytes were absent, platelets were rare, and primitive and naive cells were not seen. (A) Bone marrow smear. Bone marrow hyperplasia was extremely reduced, and no bone marrow granules and megakaryocytes were found. Red arrow: Plasma cells, Blue arrow: lymphocytes. (B) Peripheral blood smear. No platelets were found. Red arrow: lymphocytes, Blue arrow: red blood cells. (C) Histological biopsies. Red arrow: Bone trabeculae, Blue arrow: hematopoiesis.

His bone marrow evaluation showed extremely reduced bone marrow cellularity, a significantly increased ratio of granulocytes to erythrocytes, and a significantly increased proportion of mature lymphocytes in both the bone marrow (66.0%) and peripheral blood (86.0%) (Figure 1). These findings suggested the possibility of aplastic anemia. The results of lymphocyte subgroups detected by flow cytometry showed that lymphocytes accounted for 65.46% of nucleated cells. CD55 and CD59 were expressed normally on erythrocyte, granulocyte and monocyte, which excluded the diagnosis of paroxysmal nocturnal hemoglobinuria (PNH) (Babushok 2021). Comet test and mitomycin C (MMC) test were detected by Tissuebank Biotechnology Co. Ltd. (TAB) (Shanghai, China). The proportion of chromosomal aberrations detected by comet test and mitomycin C (MMC) test was less than 25%, and no mutation associated with Fanconi anemia was found in the genetic test, thus eliminating the diagnosis of Fanconi anemia (Mehta and Ebens 2002). At the same time, the child also presented with fever and infection, necessitating aggressive treatment with blood transfusions and anti‐infective therapy. To further determine the cause of the disease, genetic testing was recommended.

### 3.2 Genetic Analysis

Genes related to bone marrow failure were captured and sequenced with an average sequencing depth of 300×. Panel sequencing identified a heterozygous classical splicing variant, c.1502+1G>A, in intron 15 of the *SRP72* gene (NM_006947.4), a gene where loss‐of‐function inherited variants have been reported to be associated with BMFS1 previously. The c.1502+1G>A variant was absent from the 1000 Genomes Project and ESP6500, but has a minimal frequency in gnomAD (0.00001606) and ExAC_ALL (0.00001693). The variant has not been reported in the disease databases HGMD and ClinVar, nor in published literature databases as of this publication.

The variant was predicted to have potential impacts on splicing by SpliceAI and the online RNA Splicer tool (https://rddc.tsinghua‐gd.org/). Both software tools suggested that the variant would affect splicing. The delta score for SpliceAI was 0.98. Predictions by the RNA Splicer tool suggested two possible abnormal splice patterns: one with the insertion of 229 bp of intron 15, which could cause premature termination, and the other with exon 15 skipping, which could result in a deletion of 78 bp.

**FIGURE 2:**
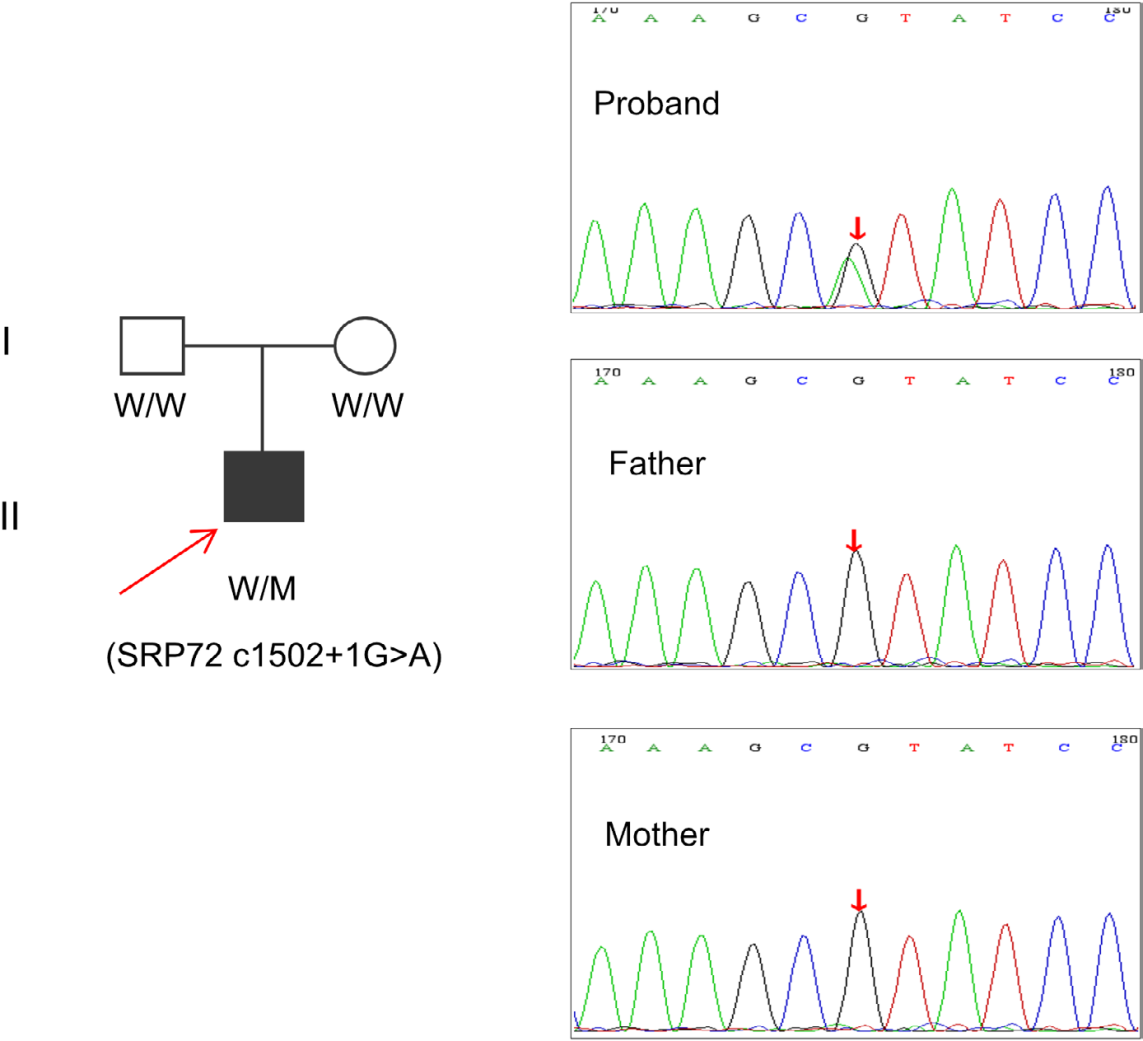
The pedigree of the family and validation of the *SRP72* gene (NM_006947.4) c.1502+1G>A mutation by sanger sequencing. Arrow indicates the proband. M, mutant type; W, wild type.

Pedigree verification by Sanger sequencing showed that neither parent carried the c.1502+1G>A variant, indicating it is a de novo variant (Figure 2).

### 3.3 Splicing Analysis by Minigene Assay

**FIGURE 3:**
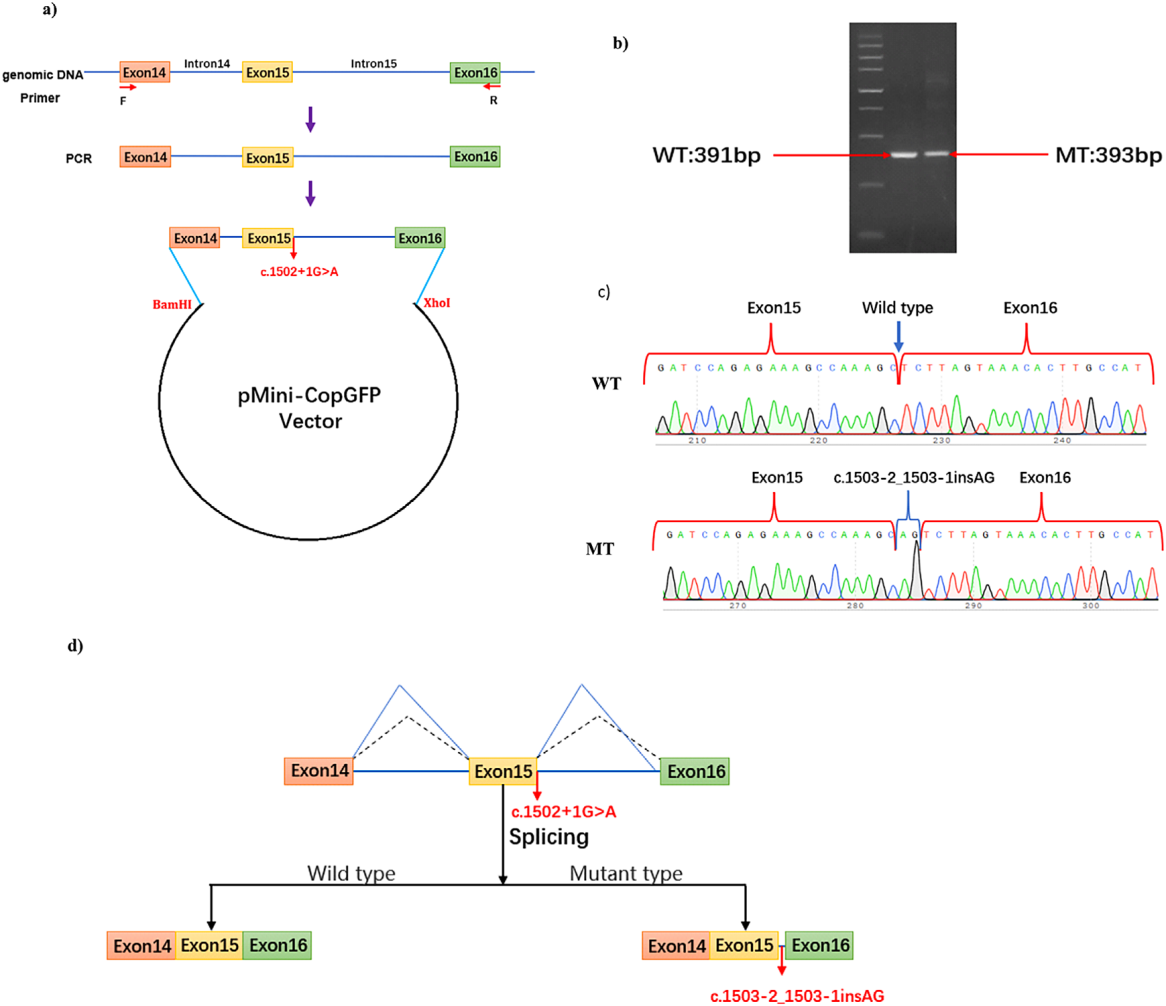
Studies on aberrant transcription of *SRP72* (NM_006947.4) c.1502+1G>A by minigene splicing assay. (a) Schematic diagram of minigene expression plasmid construction. (b) The results of the RT‐PCR products on agarose gel electrophoresis showed a single and similar‐length band in the wild‐type and mutant‐type. (c) The products of RT‐PCR were sequenced by Sanger, and the wild‐type fragment is 391 bp, the mutation type 393 bp. (d) Schematic of splicing for *SRP72* c.1502+1G>A. MT, mutation type; WT, wild type.

Minigene analysis was performed on the wild‐type and mutant types carrying *SRP72* c.1502+1G>A to further characterize the abnormal splice patterns. The results of the RT‐PCR products on agarose gel electrophoresis showed a single and similar in length band in the wild‐type and mutant type (Figure 3b). The Sanger sequencing showed that 2‐base retention of intron 15 in mutant type cDNA‐amplified products(Figure 3c). The minigene analysis showed that the c.1502+1G>A mutation generated a new splice donor site (AT) in intron 15 of the *SRP72* gene, leading to the retention of 2 bases (AG) (NM_006947.4 r.1503‐2_1503‐1insAG) (Figure 3d). This is predicted to result in a frameshift variant (p.Leu502ValfsTer14), which alters the amino acids and derives a premature termination codon. According to the ACMG guidelines, the variant was determined to be likely pathogenic (PVS1+PM6) (Richards et al. 2015; Walker et al. 2023).

## 4 Discussion

We reported a 6‐year‐old boy with aplastic anemia and pancytopenia, where panel sequencing revealed a de novo splicing variant, c.1502+1G>A, in *SRP72*, causing BMFS1. The novel mutation was shown to cause the retention of 2 bases (AG) of intron 15 and a premature stop codon, as demonstrated by a minigene assay.

The pathogenesis of the BMFS1 is not yet fully understood. *SRP72* is located on chromosome 22q11.21 and contains 19 exons. The human *SRP72* protein includes a SRP68 protein binding domain (PBD) (10–159 aa), which consists of four atypical tetratricopeptide repeats (TPR) in the N‐terminal region, as well as an RNA‐binding domain (RBD) (552–603 aa) that binds to the SRP S domain in the C‐terminal region (Gao et al. 2017). The *SRP72*‐RBD is a flexible peptide that interacts with the 5 e‐ and 5 f‐loops of SRP RNA (Iakhiaeva et al. 2010). The mutation c.1502+1G>A is predicted to result in a premature termination (p.Leu502ValfsTer14), which effectively loses the RBD domain binding to 7SL RNA.

In 2012, Kirwan et al. (2012) first reported the relationship between the *SRP72* gene and BMFS1. A family of four individuals (the mother and three siblings) with bone marrow failure and congenital nerve deafness was found to carry the *SRP72* gene mutation (c.1064_1065del, p.Thr355Lysfs*19). In this family, the mother had myelodysplasia, one sibling was diagnosed with aplastic anemia by bone marrow biopsy, and the other two siblings had pancytopenia based on full blood counts. In addition, one of 96 patients with bone marrow failure was found to have an *SRP72* gene mutation (c.620G>A, p.Arg207His), which was inherited from her mother, who also had bone marrow failure. This suggests that the mutations c.1064_1065del and c.620G>A affect SRP protein localization, as demonstrated by in vitro functional tests. Later, Bluteau et al. (2018) reported three families with *SRP72* mutations diagnosed as bone marrow failure. Among them, two cases carried pathogenic compound heterozygous mutations (c.258+2T>C and c.184A>T) in SBDS, presenting with the phenotype of acute myeloid leukemia (AML) with dysplastic malformation syndrome or bone marrow dysgenesis. Additionally, they also carried an *SRP72* gene deletion (chr4 g.57350823_57355358del) and a missense mutation (c.28T>A), respectively. The third family was a consanguineous marriage. The proband, who carried an *SRP72* gene deletion (chr4.57340035_57349299del), had neonatal transfusion dependence with malformation syndrome, and her sister died of an undiagnosed blood disease at the age of 8 months. Similuk et al. (Similuk et al. 2022) reported a heterozygous mutation of c.65G>A in the *SRP72* gene in a 65‐year‐old patient with a complex immunophenotype, suggesting susceptibility at this locus. The patient we reported is the seventh case of bone marrow failure caused by an *SRP72* gene mutation worldwide and the first case in China. In China, reports of *SRP72* gene variants are scarce and lack systematic research.

Together with the present case, there are seven published families with a *SRP72*‐related disorder. The addition of our case further supports the link between *SRP72* and BMFS1. Among all the reported families, there were 2 gross deletions, 2 truncating variations, 3 missense variations. Both of the 2 truncated variations resulted in loss of the PBD region and that possibly affects binding to SRP68. Alternatively, a complete loss of the protein due to nonsense‐mediated mRNA decay (Gardner 2010) is the cause of the phenotype. The 2 gross deletions completely contain the *SRP72* gene and also caused loss of function. Mice with heterozygous loss of *SRP72* showed mild reductions in blood and bone marrow cellularity and minor changes within the stem/progenitor compartment, but no hematological disorder was observed. Interestingly, gene expression analysis of Srp72+/− mice demonstrated that genes encoding secreted factors were transcriptionally down‐regulated (D'Altri et al. 2019). These findings provide new insights into the mechanism by which loss of *SRP72* function is associated with familial dysgenesis and myelodysplasia.

According to the reported families, common manifestations of BMFS1 with *SRP72* gene mutations include aplastic anemia, pancytopenia, myelodysplasia (Bluteau et al. 2018; Kirwan et al. 2012; Similuk et al. 2022). Physical deformities or deafness also has been observed in affected individuals (Bluteau et al. 2018; Kirwan et al. 2012). BMFS1 is characterized by early‐onset aplastic anemia or pancytopenia in some patients, and adult‐onset myelodysplasia in others. The reported case presented with progressive pancytopenia, without physical abnormalities or deafness. BMFS1 has various phenotypes and lacks highly specific clinical features, which can easily lead to misdiagnosis or missed diagnoses. Therefore, genetic testing is an effective method for diagnosing BMFS1 (Skibenes et al. 2021). Studying the *SRP72* gene mutation spectrum benefits the identification of genotype–phenotype associations and the exploration of pathogenic mechanisms.

Bone marrow failure syndromes (BMFSs) are a heterogeneous group of disorders characterized by complex overlapping phenotypes, including dysmorphogenesis, one or more extrahematopoietic abnormalities, and an increased risk of malignant transformation. More than 100 disease genes have been reported to be associated with BMFS, with major fundamental biological pathways involved including DNA repair, telomere maintenance, and ribosome biogenesis (Dokal et al. 2022; Da Costa et al. 2020; Bezzerri and Cipolli 2019). The discovery of the *SRP72* gene adds another possible biological pathway: protein translocation and processing.

Major advances in supportive treatment have led to considerable improvements in the prognosis of these patients. Supportive treatment includes red cell transfusions (typically maintained at 80 g/L), platelet transfusions (maintained at ≥ 10 × 10^9^/L), and infection prevention or anti‐infection treatment (Dokal et al. 2022; Bluteau et al. 2018). The reported patient also received symptomatic supportive treatment during the diagnostic process. Hematopoietic stem cell transplantation (HSCT) is a crucial treatment modality for patients with bone marrow failure syndromes. Among the reported cases, only two patients underwent HSCT (Sakaguchi and Yoshida 2022; Tarlock et al. 2022). One had a good prognosis, while the other failed due to acute immune rejection. After the diagnosis of BMFS1 by genetic testing, the child received hematopoietic stem cell transplantation from a sibling. After 132 days post‐transplantation, all parameters of the patient were within the normal range.

In conclusion, we reported a BMFS1 patient with an *SRP72* gene mutation, which is the first reported case in China. The mutation c.1502+1G>A had not been previously documented, and minigene studies revealed that this mutation results in abnormal splice patterns, thereby expanding the *SRP72* gene mutation spectrum. Genetic testing has become an important technique for the diagnosis of BMFS.

## Author Contributions

**Wang Xiangwen:** co‐first author and corresponding author, responsible for case diagnosis and treatment, data collection, literature review, manuscript preparation, and final approval of the version to be published. **Zhang Duo:** co‐first author, contributed to case diagnosis and treatment, literature review, and provided technical support. **Hao Wenjing:** participated in data collection, literature review, research design, and provided critical revisions for important intellectual content. **Hou Hui:** contributed to case diagnosis and treatment and provided technical support.

## Funding

This study was supported by the Health Commission of Inner Mongolia Autonomous Region (Grant 202201063). The funding body had no role in the design of the study, the collection, analysis, and interpretation of data, or in the writing of the manuscript.

## Supporting information


**Data S1:** mgg370168‐sup‐0001‐DataS1.pdf.

## Data Availability

The data that support the findings of this study are available on request from the corresponding author. The data are not publicly available due to privacy or ethical restrictions.
