# Supplementary material for: A De Novo Splicing Mutation of SRP72 in Bone Marrow Failure Syndrome Type 1: Case Report and Review of the Literature
Source: Mol Genet Genomic Med. 2025 Dec 31;14(1):e70168. doi: 10.1002/mgg3.70168 (PMC12754569; doi:10.1002/mgg3.70168)
Supplement: Supplementary file 1 — Data S1: mgg370168‐sup‐0001‐DataS1.pdf. [file MGG3-14-e70168-s001.pdf]

基因分析报告

样本编号：	22C436192	姓名：	王[redacted]	性别：	男
年龄：	6 岁 0 月	样本类型：	外周血	采样日期：	2022/12/09
送检单位：	内蒙古自治区人民医院			病历号：	-
分析项目：	H044p1：骨髓衰竭综合征 panel				
临床诊断：	-				
疾病表型：	-				
重点关注基因：	-				
病史摘要：	重点分析：先天性骨髓衰竭				
家族史摘要：	-				
检测方法：	王[redacted]高通量测序				

家系临床信息：

样本标记	样本关系	样本编号	标准化后的 HPO 表型或疾病
王[redacted]	本人	22C436192	骨髓细胞过少,骨髓增生异常
王[redacted]父亲	父亲	22C617795	-
王[redacted]母亲	母亲	22C617794	-

分析结果：

通过对疾病相关基因的测序分析，发现与疾病表型相关的高度可疑变异

一、 临床表型高度相关，且致病性证据较为充分的基因变异：

基因	染色体位置	转录本外显子	核苷酸氨基酸	纯合/杂合	正常人频率	预测	ACMG 致病性分析	疾病/表型(遗传方式)	变异来源
SRP72	chr4:57356812	NM_006947.4;intron15	c.1502+1G>A (p.?)	het	0.0000169	-	Pathogenic	骨髓衰竭综合征 1 型(AD)	自发

注：预测：蛋白功能预测软件 REVEL, D:预测为有害;LD:预测为可能有害;U:预测为不确定;LB:预测为可能良性;B:预测为良性; -:未知

基因变异信息概述：发现*SRP72*基因有1个杂合突变。c.1502+1G>A(p.?)经家系验证分析，受检人之父该位点无变异，受检人之母该位点无变异。

1）基因变异信息详细解读：

该样本分析到 *SRP72* 基因有 1 个杂合突变：c.1502+1G>A 杂合突变，导致氨基酸发生剪接突变（p.?）。ACMG 遗

传变异信息详细解读如下：

(1)c.1502+1G>A（intron15，NM_006947.4），导致氨基酸改变 p.?，为剪接突变。
根据 ACMG 指南，该变异初步判定为致病性变异（Pathogenic）PVS1+PS2：
- PVS1：该变异为零效变异（剪接突变），可能导致基因功能丧失；
- PS2：经家系验证分析，受检人之父该位点无变异，受检人之母该位点无变异，此变异为自发突变 ；

文献数据库未有该位点的相关性报道 ，ClinVar 数据库无该位点致病性分析结果；在正常人群数据库中的频率为 0.0000169；

2）疾病临床描述：

基因： *SRP72*

疾病：

(1)骨髓衰竭综合征 1 型（OMIM：614675）；AD

疾病介绍： 骨髓衰竭综合征 1 型的主要特征包括再生障碍性贫血和脊髓发育不良。再生障碍性贫血是一种骨髓无法制造出足够数量的外周血成分而引起的贫血。脊髓发育不良是一种克隆造血干细胞紊乱，骨髓中的未成熟细胞出现畸形和功能失调。

临床表型： 听力障碍；再生障碍性贫血；骨髓增生异常；骨髓细胞过少；全血细胞减少症；儿童期发病

临床表现相关文献： Kirwan 等(2012)报道了一个家庭，其中 3 个同胞，年龄在 11 到 14 岁之间，患有早发性再生障碍性贫血或全血细胞减少症，他们的母亲患有骨髓发育异常。均有先天性神经性耳聋。没有人接受血液异常治疗。在另一个家庭中，母亲和女儿都患有成人发病的骨髓发育异常。两个人都没有耳聋，但女儿可能得了迷路炎。既不接受治疗。

临床建议： 以上结论均为基因分析结果，仅供参考，请结合受检者的临床表现、家族史及其他检测结果综合分析。

质控数据统计：

受检者	样本编号	平均测序深度	目标区域覆盖度	
			10X	20X
王[illegible]	22C436192	325.59	98.65	97.67

方法学：

测序分析

局限性：

1. 本检测项目只针对已知的与疾病相关的基因，一些尚未明确的基因不在检测范围内。
2. 在数据分析时，为保证数据分析的精确性，目标区域内少部分测序质量过低的变异将被滤掉。鉴于当前医学检测技术水平的限制和受检者个体差异等原因，本检测无法保证 100%的准确性以及 100%的成功率。该方法适用于所送检基因的外显子区（不包括启动子区等非编码区）及外显子相邻 20bp 的内含子区中的点突变（检测准确率为 99%以上）、小的缺失插入突变（20bp 以内）（检测准确率为 99%以上）。
3. 对在捕获区域内的突变，如其所在区域为高 GC 含量区，高度重复序列区，或者在基因组其它位置存在高度同源序列，本方法可能存在一定的假阴性几率。部分基因存在高重复低复杂度区域或假基因，以致检测不能完全覆盖其所有外显子区，但总体覆盖度可达 95%以上。

受捕获效率影响，不同基因及外显子区段的覆盖度可能无法达到 100%。调控区及深度内含子区可能存在的致病性变异无法检测。
4. 此检测方法可以分析到小片段缺失/插入变异，对于更大范围内的缺失/重复，也可以分析，但可信度会降低，需要另一种方法验证，可提供参考结果；NGS 无法检测出所有拷贝数变异，如果怀疑基因存在拷贝数变异，可以进行针对性的 aCGH、SNP array 或 MLPA 分析。此外，未进行 NGS 测序的家系样本不提供缺失重复结果。
5. 本方法不适于检测特殊类型变异（包括但不限于体细胞突变、深度内含子变异、动态突变、基因甲基化、假基因区域变异、复杂重组等，上述情形均为不适用于高通量检测的技术局限性范畴）。
6. 限于目前人类对疾病认识水平的局限性，如未检出能完全解释受检者临床表型的特定基因及致病变异位点，并不能排除受检者存在某种遗传疾病的可能性，因为某些疾病的发病可能与其它未知基因或本方法难以检测到、或无法确定的基因变异类型有关。
7. 实验室无法对医生或受检者提供的临床表型及临床怀疑疾病的真实性、准确性、全面性做出保证。由于临床表型和家族史提供不准确或不完善的样本，存在报告位点提报不精准的风险。特定变异的遗传性是基于向实验室描述的家庭关系，非母（源）性或非父（源）性的可能性未被排除。

注：
1. 如果受检者检测到了自发变异位点，该种情况下并不排除父母生殖腺镶嵌体突变的情况存在，建议遗传咨询。
2. 参考基因组版本为 GRCh37/hg19 ，核苷酸改变的位置信息是基于当前版本的基因组和转录本，不同基因组版本或不同转录本，这些信息可能会有所不同。
3. hom/het/hemi：hom 表示此突变位点为纯合突变，het 表示此突变位点为杂合突变，hemi 表示此突变位点为半合子突变。
4. MAF：正常人群频率 1000 genomes（千人基因组）、ESP6500（NHLBI Exome Sequencing Project）、EXAC（The Exome Aggregation Consortium）和 EXAC-EAS（EXAC 约 4000 东亚人数据）。
5. 蛋白功能预测软件
REVEL(rare exome variant ensemble learner) : Am J Hum Genet. 2016 Oct 6;99(4):877-885.[PMID: 27666373]，D:预测为有害;LD:预测为可能有害; U:预测为不确定;LB:预测为可能良性 B:预测为良性; -:未知。
6. 根据美国医学遗传学与基因组学会（ACMG）发布的变异解读指南进行致病性分析：pathogenic/致病性变异；likely pathogenic/疑似致病性变异；uncertain/临床意义未明变异；likely-benign/疑似良性变异；benign/良性变异。本检测只报告根据 ACMG 分级为"致病"、"疑似致病"、"临床意义未明"的变异，不报告"良性"及"疑似良性"等不具有临床意义的变异。有明确家族史的病人，如果有相关家属的基因检测结果会更有助于定性疾病相关的突变。
7. 遗传方式：AR 表示常染色体隐性遗传；AD 表示常染色体显性遗传；XR 表示 X 染色体隐性遗传；XD 表示 X 染色体显性遗传。
8. 基因核苷酸变异的解释是基于目前对该基因的了解，随着研究的深入，这些解释也可能会发生改变。对于某些目前未知临床意义的变异或与其他疾病有关的变异未列在该报告中。

*本报告结果只对送检样品负责。
*以上结论均为实验室分析数据，仅供参考。
*本公司对以上分析结果保留最终解释权，如有疑义，请在收到结果后的 5 个工作日内与我们联系。

实验人员： 张晴　　　　报告签发： 李淑葵

分析人员： 高雅君　　　　报告日期： 2023-02-02

北京迈基诺基因科技股份有限公司医学检验所 检验报告专用章 110105249573

附录1: 基因检测列表

ABCG5	*ABCG8*	*ABL1*	*ABL2*	*ACD*	*ACP5*	*ACSL6*	*ACTN1*
ADA	*ADA2*	*ADAMTS13*	*ALAS2*	*ANKRD26*	*ARHGEF1*	*ARID2*	*ASXL1*
ATG2B	*ATM*	*ATP5IF1*	*ATRX*	*BCL2L1*	*BCOR*	*BCORL1*	*BLM*
BOD1L1	*BRAF*	*BRCA1*	*BRCA2*	*BRCC3*	*BRINP3*	*BRIP1*	*CALR*
CASP10	*CBL*	*CBLB*	*CBLIF*	*CD3G*	*CDC42*	*CDKN2A*	*CEBPA*
CENPS	*CENPS-CORT*	*CENPX*	*CHEK2*	*CIITA*	*CREBBP*	*CSF3R*	*CTC1*
CTCF	*CTLA4*	*CTNNA1*	*CUX1*	*CXCR4*	*CYCS*	*DAXX*	*DCLRE1B*
DDX41	*DIAPH1*	*DKC1*	*DNAJC21*	*DNMT3A*	*DRG1*	*EED*	*EFL1*
EIF6	*ELANE*	*ELAVL1*	*EP300*	*EPCAM*	*EPO*	*ERCC4*	*ERCC6L2*
ESCO2	*ETV6*	*EZH2*	*FAAP100*	*FAAP20*	*FAAP24*	*FAN1*	*FANCA*
FANCB	*FANCC*	*FANCD2*	*FANCE*	*FANCF*	*FANCG*	*FANCI*	*FANCL*
FANCM	*FAS*	*FASLG*	*FBXW7*	*FLI1*	*FLT3*	*FMC1-LUC7L2*	*FOXP3*
FYB1	*G6PC3*	*GALE*	*GAR1*	*GATA1*	*GATA2*	*GATA3*	*GFI1*
GFI1B	*GGCX*	*GNAS*	*GNB1*	*GNE*	*GP1BA*	*GP1BB*	*GP9*
GRK3	*GSKIP*	*GTPBP4*	*HAX1*	*HJV*	*HOXA11*	*IDH1*	*IDH2*
IFNG	*IKZF1*	*IL2RA*	*IL2RB*	*IL7R*	*IRF1*	*ITGA2*	*ITGA2B*
ITGB3	*ITPA*	*JAGN1*	*JAK2*	*JAK3*	*JARID2*	*KDM6A*	*KIT*
KMT2A	*KMT2C*	*KRAS*	*LSG1*	*LUC7L2*	*MAD2L2*	*MAP2K1*	*MASTL*
MBD4	*MDM4*	*MECOM*	*MECOM-AS1*	*MLH1*	*MPIG6B*	*MPL*	*MRE11*
MSH2	*MSH6*	*MYB*	*MYBL2*	*MYC*	*MYH9*	*MYSM1*	*NAF1*
NBEAL2	*NBN*	*NCOR2*	*NF1*	*NHP2*	*NIPBL*	*NLRP1*	*NMD3*
NOP10	*NOTCH1*	*NPM1*	*NRAS*	*ORAI1*	*PALB2*	*PARN*	*PAX5*
PCNT	*PDGFA*	*PDGFB*	*PDS5B*	*PHF6*	*PMS2*	*PNP*	*POT1*
PRF1	*PRKACG*	*PRKCD*	*PRPF40B*	*PRPF8*	*PSMB6*	*PTEN*	*PTPN11*
PTPRJ	*RAD21*	*RAD51*	*RAD51C*	*RAG1*	*RAG2*	*RASGRP1*	*RB1*
RBBP6	*RBM8A*	*RFWD3*	*RFX5*	*RFXANK*	*RFXAP*	*RIT1*	*RPL10*
RPL10A	*RPL11*	*RPL12*	*RPL13*	*RPL13A*	*RPL14*	*RPL15*	*RPL17*
RPL17-C18orf32	*RPL18*	*RPL18A*	*RPL19*	*RPL21*	*RPL22*	*RPL23*	*RPL23A*
RPL24	*RPL26*	*RPL27*	*RPL27A*	*RPL28*	*RPL29*	*RPL3*	*RPL30*
RPL31	*RPL32*	*RPL34*	*RPL35*	*RPL35A*	*RPL36*	*RPL36A*	*RPL36A-HNRNPH2*
RPL36AL	*RPL37*	*RPL37A*	*RPL38*	*RPL39*	*RPL39L*	*RPL3L*	*RPL4*
RPL41	*RPL5*	*RPL6*	*RPL7*	*RPL7A*	*RPL8*	*RPL9*	*RPLP0*
RPLP1	*RPLP2*	*RPS10*	*RPS11*	*RPS12*	*RPS13*	*RPS14*	*RPS15*
RPS15A	*RPS16*	*RPS17*	*RPS18*	*RPS19*	*RPS19BP1*	*RPS2*	*RPS20*

RPS21	*RPS23*	*RPS24*	*RPS25*	*RPS26*	*RPS27*	*RPS27A*	*RPS27L*
RPS28	*RPS29*	*RPS3*	*RPS3A*	*RPS4X*	*RPS4Y1*	*RPS4Y2*	*RPS5*
RPS6	*RPS6KA1*	*RPS6KA2*	*RPS6KA3*	*RPS6KA4*	*RPS6KA5*	*RPS6KA6*	*RPS6KB1*
RPS6KB2	*RPS7*	*RPS8*	*RPS9*	*RPSA*	*RSL24D1*	*RTEL1*	*RUNX1*
SAMD9	*SAMD9L*	*SBDS*	*SETBP1*	*SF1*	*SF3A1*	*SF3B1*	*SH2B3*
SLC34A1	*SLFN14*	*SLX4*	*SMC1A*	*SMC3*	*SPRED1*	*SRC*	*SRP54*
SRP72	*SRSF2*	*STAG2*	*STAT1*	*STAT3*	*STIM1*	*STN1*	*STON1*
STON1-GTF2A1L	*SUZ12*	*TBPL1*	*TERC*	*TERF1*	*TERT*	*TET2*	*THPO*
TINF2	*TLR8*	*TNFSF13B*	*TP53*	*TPM4*	*TPP1*	*TRPM7*	*TSR2*
TTC7A	*TUBB1*	*U2AF1*	*U2AF2*	*UBA52*	*UBE2T*	*UHRF1*	*UMODL1*
USB1	*USP1*	*VPS13B*	*VPS45*	*VWF*	*WAS*	*WDR48*	*WRAP53*
WRN	*WT1*	*XRCC2*	*ZAP70*	*ZCCHC8*	*ZFPM1*	*ZRSR2*	*ZSWIM4*

附录2:　家系分析结果展示

注：由于 Sanger 验证采用正向测序或反向测序，峰图显示的碱基有可能为被检测碱基的反向互补序列，如：c.163G>A，峰图可显示为 G>A 或其反向互补序列 C>T。

分析样本	分析结果	SRP72	chr4:57356812	c.1502+1G>A	p.?
王[illegible] 22C436192	杂合变异	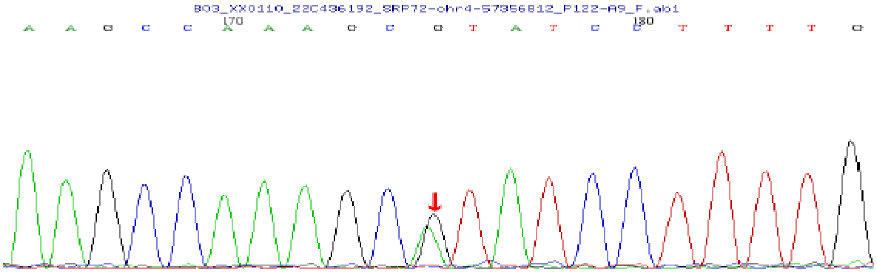			
王[illegible]父亲 22C617795	无变异	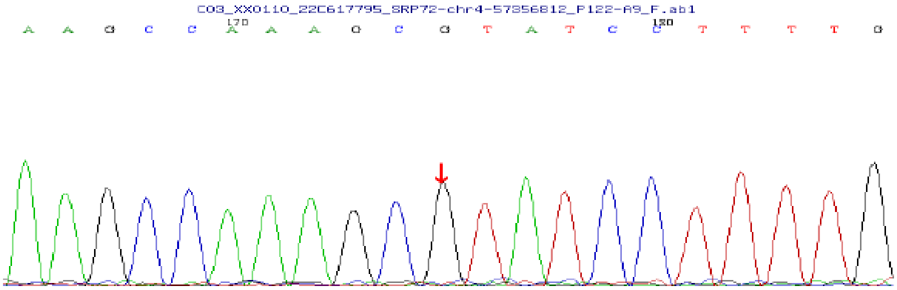			
王[illegible]母亲 22C617794	无变异	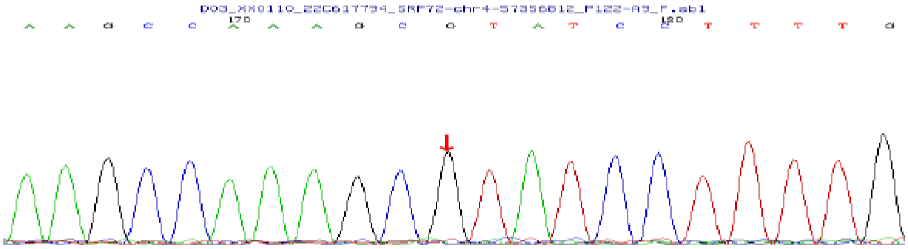			
